# Multiple disparities in adult mortality in relation to social and health care perspective: results from different data sources

**DOI:** 10.1186/s12992-017-0283-z

**Published:** 2017-08-08

**Authors:** Chhabi Lal Ranabhat, Chun-Bae Kim, Myung-Bae Park, Sambhu Acharaya

**Affiliations:** 1 Department of Preventive Medicine, Yonsei University Wonju College of Medicine, Wonju, Gangwon-do 26426 Republic of Korea; 2 Institute for Poverty Alleviation and International Development, Yonsei University, Wonju, Ganwon 26493 Republic of Korea; 3 Health Science Foundations and Study Center, Kathmandu, Nepal; 4 Department of Country Cooperation and Collaboration with the UN System Office of the Director-General, World Health Organization, Geneva, Switzerland; 5 Department of Gerontal Health and Welfare, Pai Chai University, Seo-gu, Daejeon, Republic of Korea

**Keywords:** Adult mortality, Disparity, Political instability, Universal health coverage, Cross-country study

## Abstract

**Background:**

Disparity in adult mortality (AM) with reference to social dynamics and health care has not been sufficiently examined. This study aimed to identify the gap in the understanding of AM in relation to religion, political stability, economic level, and universal health coverage (UHC).

**Methods:**

A cross-national study was performed with different sources of data, using the administrative record linkage theory. Data was created from the 2013 World Bank data catalogue by region, The Economist (Political instability index 2013), Stuckler David et al. (Universal health coverage, 2010), and religious categories of all UN country members. Descriptive statistics, a t-test, an ANOVA followed by a post hoc test, and a linear regression were used where applicable.

**Result:**

The average AM rate for males and females was 0.20 ± 0.10 and 0.14 ± 0.10, respectively. There was high disparity of AM between countries with and without UHC and between groups with low and high income. UHC and political stability would significantly reduce AMR by >0.41 in both sexes and high economic status would reduce male AMR by 0.44, and female AMR by 0.70.

**Conclusions:**

It can be concluded that **e**ffective health care; UHC and political stability significantly reduce AM.

## Background

Adults, aged between 15 and 60 years are the pillars of economic growth and national development [1]. The death of adults significantly influences the mortality and morbidity in others, like infants, children, and mothers, as well as the health and education of the children in their family [2–4]. The twentieth century has witnessed a surge in adult mortality (AM) [5]. There is a huge disparity in the mortality rates among different age groups; for example, the global AM rate is 123 times higher than maternal mortality, 8 times higher than infant mortality, and 6 times higher than the mortality rate in children younger than 5 years [6]. Likewise, the African country of Lesotho has the highest male AM rate (0.58), which is 10 times higher than that of the European country of Sweden (0.06), which has the lowest male AM rate [7]. Similarly, female AM rate is the highest in Swaziland (0.61), which is 20 times higher than that of Cyprus (0.03) [8]. The World Health Report 2014 shows that Africa has a higher AM than other regions and it could be due to the economic inequality [9]. There has been a large amount of research, discussion, interpretation, programs development and policies for child and maternal mortality, but there are no specific targets or policies to mitigate AM since the origin of “Primary Health Care” approach to current UN “Sustainable Development Goal (SDG).” To explain adult deaths, medical causes and demographic factors have been explored [10]. However, factors related social dynamics and the health care system are rarely discussed.

Adult death is affected by numerous factors such as the high mobility, frequently exposure to risky occupations, social conflict, and unhealthy lifestyle [11]. Previous research highlighted the risk factors and pathological conditions that affect AM. There are high magnitudes of research on age specific mortality by diseases, injury and events like cancer [12], road traffic injuries [13], TB/HIV [14], reproductive health problems (maternal mortality) [15], risk behaviors [16] etc. by Institute for Health Metrics and Evaluation [17] on global burden of disease which are the visible causes of death. Communicable disease and injury (49%) and non-communicable disease (51%) were the direct (medical) causes of AM in Ethiopia [18]. Richard G Rogers and T Pensola et al. reported that socio-behavioral factors like conflict, unemployment, poverty, and cultural and religious violence make adults more vulnerable to death [19, 20]. Li Quan and Ming Wen stated that political conflict directly increases male AM and has a long term psychological effect on female [21]. In his long term research, Jonathan Fox found that religious conflict also increases AM [22]. Ethnic, political, and religious conflict increased the incidence of injury and death in adults in Northern Kosovo [23]. Beyond these social dynamics, the health policy and health care management system are mainly responsible in predicting AM. After the effective implementation of a health care management policy and cessation of civil war in Sri Lanka, adult death has been explicitly reduced [24]. The Power of Prevention by the Center for Diseases Control and Prevention (CDC) 2009 concluded that chronic diseases are mainly responsible for AM and that a comprehensive health policy like Universal Health Coverage (UHC) could improve health outcomes and reduce all kinds of mortality including that in adults [25].

**Fig. 1:**
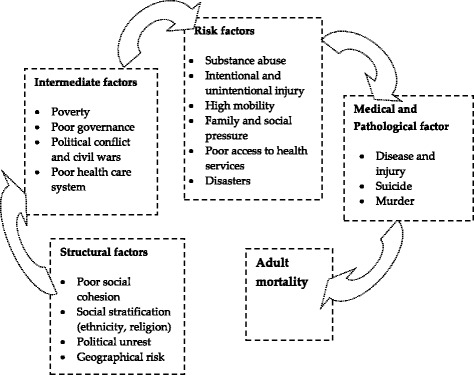
Conceptual figure of adult mortality with multiple dimensions

Thus, there are multiple dynamics in AM. Sartarious et al. explored the long-term, mid-term, and short-term aspects of AM and categorized social characteristics; policy intervention as long-term, health care system characteristics as medium-term, and individual characteristics as short-term factors [26]. The Commission of Social Determinants of Health by the World Health Organization (WHO) indicated that the socioeconomic and political contexts are the structural factors responsible for health, disease, and mortality, while the health care system and policy characteristics are intermediate factors [27]. Due to age, family and social responsibilities, adults incur unique risks and are at high risk for injuries, social deviation, risky job placement, and vulnerability for premature death [28]. Based on the findings of the above studies, a conceptual framework was developed to include multiple factors related to AM (Fig. 1). The framework categorized 4 blocks for AM: structural, intermediate, risk, and pathological/medical factors. The social and political environments of an individual are structural factors; poverty, poor governance, and health care system characteristics are intermediate factors; individual characteristics are risk factors; and direct causes of death are medical or pathological factors. To maintain the social equity, it is necessary to adopt effective social policy, like poverty alleviation approach, health care to the needy people, good governance and social harmony program to minimize conflict. Such approach is the permanent way to balance structural factors and significantly reduces adult mortality. With regard to AM, research related to structural factors is very rare, that on the intermediate factors is uncommon, while that regarding risk and medical factors is much more prevalent.

As previously discussed, multiple factors impact AM; therefore, highlighting a single factor is insufficient. It is necessary to examine the impact on AM in combination with social dynamics like economics, politics, religion, and comprehensive health policy, and the mortality gap between different clusters. Past global and national comparisons, such as those conducted by the World Health Organization (WHO), World Bank (WB), Organization of Economic Cooperation and Development (OECD), and others, have sufficiently explored the gap between different regions*.* They have continuously concluded that the disparities among mortality are increasing in Africa (geographical) and among poor people (economic). Beyond the biological and economic factors, it is necessary to identify the social environment factors, such as political conflict, religion, and existing health care management policy, related to adult death. In other words, some adult deaths due to biological factors can be prevented within a few years; risk factors can be controlled in the presence of a good health care system within a decade. However, death due to social causes like conflict and political unrest cannot be controlled even in hundreds of years. After exploring the multiple disparities, it is easy to figure out the problems in policies and programs. In this study, we explored the distribution of AM based on social domains and health care system to highlight the inequalities in AM using different sources of data from all possible UN member states. This is important because there has been significant progress in other areas of morbidity and mortality; however, the AM pattern has remained unchanged for several decades, and it demands special interventions and approaches. Our study explores the socio-structural aspects of adult mortality, which is little far from causal relation of diseases or mortality. This study aimed to identify the gaps in male and female AM in different clusters of countries in terms of their region, economic level, political stability, religion, and UHC.

## Methods

This was a secondary data-based cross-national study of UN member states (193), conducted on data from 2012/13, from multiple sources. Data were sourced from open access internet subscribed systems.

### Data adoption theory

Data were created based on the Record Linkage Theory proposed by Halbert L Dunn in 1946 [29]. The objective of this model was to preserve the records, verify them, and create new statistics from various sources of data, even though the data could have been collected for different purposes. Furthermore, this model was advanced by Douglas P. Jutte, 2011, as an Administrative Record Linkage tool for public health research [30]. Based on this theory, the data were created from multiple sources.

### Data sources

Table 1 shows the open access sources of data used for this study*.*

**Table 1:** Sources of data and related reference

Source of complete data set	Reference
World Bank (Adult mortality for males and females) 2012/13	World Bank. Data Catalogue; 2013 [6]
Category of countries that adopted and achieved universal health coverage	Stuckler D, Feigl AB, Basu S, McKee M. The political economy of universal health coverage; 2010 [50]
Categories of countries of different religions	Barro RJ, Mc Cleary RM. Which countries have state religions?: National Bureau of Economic Research; 2004 [51]
Categories of countries of different economic levels	World Bank. Country income group; 2016 [52]
Categories of countries of different geographical regions	World Bank. Data Catalogue; 2013 [6]
Categories of countries based on political stability	The Global Economy. Political Stability Index; 2013 [53]

### Data categorization

After importing the data from the different sources shown in Table 1, the independent variables were categorized. The categories of geographical location were Asia Pacific: 1, Europe and Central Asia: 2, Latin America and Caribbean: 3, Middle East and North Africa: 4, South Asia: 5, Sub-Saharan Africa: 6, and North America: 7. Likewise, economic level, religion, and status of UHC were categorized with numerical labels. The Global Economy published the political stability index and has categorized countries on a scale of +2.5 to −2.5. We used this index to create three categories of ≥1.0 as stable, 0.0–1.0 as average, and <−0.01 as unstable countries.

### Data management

The country-wise data were prepared in Microsoft Excel for all the 193 UN members, from the above sources. Data and outliers were cross checked and missing data from some countries were excluded from the study. After verification and re-verification, the database was exported into the Statistical Package for Social Sciences (SPSS) ver. 21.0 by IBM (SPSS Inc., Chicago, IL, USA). A *p*-value of less than 0.05 was considered statistically significant.

### Data analysis model

1. The descriptive results were presented in terms of frequencies and percentages for different clusters of countries.
2. In the second phase, an independent t-test and analysis of variance (ANOVA) followed by a post hoc analysis were used to examine the mean differences.
3. In the third phase, a linear regression was used after converting the categorical variables into dummy variables. The dummy variables were created as n-1 from the total categories in each group. Among 5 groups, we selected 3 variables; political stability, economic status, and UHC. For the regression, religion and geographical region were not included because they showed no theoretical foundation and would influence possible interpretation bias with regard to AM.

### Variables

The different categorical variables were chosen as independent variables according to the research concept and model. The categories were 7 global geographical locations, 5 economic levels, 3 statuses of political stability, 5 categories of religion, and 2 statuses of UHC (yes/no). AM in per thousand males and females was the dependent variable.

### Validity and reliability

There are very few questions about the validity and reliability of the Word Bank and related data sources. The same data have been used in this study. Sources of data were available from their home pages and were easy to access. After re-entry of the different sources of data, cross checking and confirmation was done at least twice or more if needed. Likewise, the normality of the data was verified by observation of the histogram, and consistency of the data was checked by the Cronbach’s alpha for appropriate variables.

### Ethical approval

Ethical approval was not considered necessary because the data sources are open access, available from web pages and published articles.

## Result

### Descriptive statistics

The number of countries was not always the same in each group. Out of the 5 categories, political stability data were available for 191 countries (Table 2). Data showed that about 1/4th these countries were located in Sub-Saharan Africa and only 2 countries belonged to North America. More than half of the countries were categorized as low and middle-income countries, and in half of countries, there is political instability, no state religion, and only about 1/3rd countries have achieved UHC. The average AM rates for men and women were 0.20 ± 0.10 and 0.14 ± 0.10, respectively.

**Table 2:** Descriptive statistics by country categories

Characteristics	Categories of countries	Frequency	%
Geographical region (*N* = 193)	Asia Pacific	30	15.51
	Europe and Central Asia	51	26.41
	Latin America and Caribbean	35	18.11
	Middle East and North Africa	19	9.83
	South Asia	8	4.12
	Sub-Saharan Africa	48	24.91
	North America	2	1.01
Economic level (*N* = 193)	Low income	32	16.62
	Lower middle income	49	25.41
	Upper middle income	51	26.42
	High income: Non OECD	29	15.03
	High income: OECD	32	16.62
Political stability (*N* = 191)	Instability	91	47.64
	Average	69	36.12
	Stable	31	16.23
Religion (*N* = 193)	No state religion	95	49.23
	Christian	53	27.51
	Muslim	30	15.52
	Buddhist	7	3.63
	Other traditional religion	8	4.11
Achievement of universal health coverage (*N* = 193)	No	137	70.98
	Yes	56	29.02

The data were considered reliable based on the Cronbach’s alpha value of 0.724.

### Comparison of adult mortality between and within groups

The following tables show the multiple disparities in AM. There was a significant difference (*p* < 0.05) between male and female AM in all groups, i.e., geographic region, economic level, political stability, religion, and presence of UHC.

The male AM rate was the highest in Sub-Saharan Africa (0.31), low income countries (0.31), politically unstable or conflict-ridden countries (0.25), countries with a traditional religion (0.31), and countries without UHC (0.24) (Table 3). Comparing the means of the groups using the Bonferroni post-hoc analysis, male AM was significantly higher (*p* < 0.001) in Sub-Saharan Africa than in the Asia Pacific, Europe and Central Asia, Latin America and Caribbean, Middle East and North Africa, South Asia and North Africa regions. Likewise, male AM was significantly higher in low-income countries than in high-income countries, in politically unstable countries than in average or stable countries, and in countries with a traditional religion. The countries with UHC had significantly reduces male AM than did those without. The difference in the male AM rates between Sub-Saharan and other geographical regions, between low income countries and other economic categories, between politically unstable countries and average or stable countries, between countries with no state religion and those which were Christian, Muslim or other traditional religion countries, and between countries with and without UHC was also statistically significant (*p* < 0.05). The F statistic was the highest in the UHC category (61.89) and lowest in the religion category (3.63).

**Table 3:** Male adult mortality rate per thousand individuals by different country categories

Characteristics	Category	Mean ± SD	Mean Difference	*p* value	F value
Geographical region	Sub-Saharan Africa (Ref.)	316.77 ± 97.49		< 0.001	19.11
	Asia Pacific	175.40 ± 71.59	141.37^**^		
	Europe and Central Asia	165.14 ± 90.09	151.62^**^		
	Latin America and Caribbean	198.30 ± 72.34	118.47^**^		
	Middle East and North Africa	136.05 ± 69.29	180.71^**^		
	South Asia	197.37 ± 59.62	119.39^**^		
	North America	106.50 ± 33.23	210.27^**^		
Economic level	Low income (Ref.)	319.90 ± 77.73		< 0.001	36.33
	Lower middle income	257.02 ± 89.47	62.87^*^		
	Upper middle income	196.97 ± 85.04	122.92^**^		
	High income: Non OECD	149.76 ± 87.92	170.14^**^		
	High income: OECD	102.96 ± 38.10	216.93^**^		
Political stability	Unstable (Ref.)	254.2 ± 96.4		< 0.001	23.72
	Average	176.6 ± 107.3	77.58^**^		
	Stable	98.8 ± 32.7	155.39^**^		
Religion	No state religion (Ref.)	225.5 ± 102.8		0.046	3.63
	Christian	186.9 ± 101.6	38.58^*^		
	Muslim	180.0 ± 97.2	45.56^*^		
	Buddhist	197.6 ± 74.6	27.90		
	Other traditional religion	310.0 ± 149.3	−84.43^*^		
Universal Health Coverage	No (Ref.)	244.5 ± 100.3		< 0.001	61.89
	Yes	126.5 ± 66.2	118.0**		

Ref.: Reference; *p < 0.05, **p < 0.01

A trend similar to that observed in male AM was seen in terms of female AM, but the magnitude was slightly different. Table 4 shows that female AM was the highest in low-income countries, Sub-Saharan Africa, countries with political conflict, absence of UHC, and a traditional religion. A post-hoc test (Bonferroni) showed that female AM was significantly higher (*p* < 0.001) in low-income countries than in lower middle-income, upper middle-income, high-income non-OECD, and high-income OECD countries. Similarly, female AM was significantly higher in Sub-Saharan Africa than in other regions, in politically unstable countries than in average or stable countries, and in countries with a traditional religion. The countries with UHC had significantly lower female AM than did those without. As observed in terms of male AM (Table 3), the differences in female AM rate between Sub-Saharan and other geographical regions, between low income countries and other economic categories, between politically unstable countries and average or stable countries, between countries with no state religion and those which were Christian, Muslim, or had any other traditional religion, and between countries with and without UHC was also statistically significant (*p* < 0.05). The F statistic was the highest for the UHC category (51.85) and lowest for the religion category (4.31).

**Table 4:** Female adult mortality per thousand individuals by different country categories

Countries group	Category	Mean ± SD	Mean difference	*p* value	F value
Geographical region	Sub-Saharan Africa (Ref.)	265.70 ± 106.77		< 0.001	28.66
	Asia Pacific	113.44 ± 58.40	152.26833^**^		
	Europe and Central Asia	83.22 ± 54.72	182.48384^**^		
	Latin America and Carrabin	124.33 ± 72.85	141.37500^**^		
	Middle East and North Africa	92.42 ± 56.61	173.28728^**^		
	South Asia	150.37 ± 62.84	115.33333^**^		
	North America	64.50 ± 17.67	201.20833^**^		
Economic level	Low income (Ref.)	280.20 ± 82.99		< 0.001	42.39
	Lower middle income	179.84 ± 101.71	100.35217^**^		
	Upper middle income	122.28 ± 71.72	157.91429^**^		
	High income: Non OECD	87.280 ± 63.59	192.92000^**^		
	High income: OECD	52.67 ± 12.20	227.52258^**^		
Political stability	Unstable (Ref.)	178.8 ± 106.6		< 0.001	13.74
	Average	120.0 ± 100.4	58.75487^*^		
	Stable	54.2 ± 13.8	124.52197^**^		
Religion	No state religion (Ref.)	158.7 ± 106.4		0.012	4.31
	Christian	126.5 ± 99.0	32.27147		
	Muslim	116.6 ± 79.1	42.10104		
	Buddhist	123.0 ± 64.7	35.79070		
	Other traditional religion	266.1 ± 147.9	−107.33430^*^		
Universal Health Coverage	No (Ref.)	178.3 ± 106.2		< 0.001	51.85
	Yes	69.0 ± 45.6	109.3**		

Ref.: Reference; *p < 0.05, **p < 0.01

### Regression analysis between variable groups and adult mortality

Two separate multivariate analyses (male and female AM) were performed using linear regression. Three independent variables (economic level, political stability, and UHC) and AM rate (male and female) were included in the regression to examine the magnitude of association. In the regression, the variables in the model could explain 46% and 48% (adjusted R^2^ = 0.46 and 0.48) of the variance in male and female AM rate, respectively.

Having UHC would significantly reduce (*p* < 0.01) AM rate by 0.42 and 0.41 for males and females as compared to not having UHC. Likewise, countries with adequate political stability and average political stability would have lower AM rate by 0.40 and 0.16 for males and 0.41 and 0.17 for females as compared to countries with political instability. A similar trend was observed with economic level. Countries with high income: OECD could have a lower AM rate by 0.44 and 0.70 for males and females, respectively, in comparison to low economic strength countries. The economic strength would reduce the AM rate for female almost twice the extent of reduction in male AM rate (Table 5).

**Table 5:** Relation between UHC, political stability, and economic sub-categories with adult mortality rate

Independent variables	Male Adult Mortality		Female Adult Mortality	
	Stand.coff.	*p* value	Stand.coff.	*p* value
(UHC No ref.)				
UHC Yes	−.424	< 0.001	−.412	< 0.001
(Unstable ref.)				
Average Political stability	−.166	0.028	−.179	0.021
Political stable	−.416	0.005	−.414	< 0.001
(Low income ref.)				
Lower middle income	−.255	0.001	−.414	< 0.001
Upper middle income	−.279	< 0.001	−.636	< 0.001
High income: Non OECD	−.380	< 0.001	−.582	< 0.001
High income: OECD	−.443	< 0.001	−.705	< 0.001
R^2^	0.48		0.50	
Adjusted R^2^	0.46		0.48	
*P* value	< 0.001		< 0.001	
F value	23.36		24.76	

Stand. Coff.: Standardized coefficient

## Discussion

This study showed the different social aspects of AM gap, particularly, those related to political stability, religion, economic strength, achievement of UHC, and the geographical region of countries. Our study showed global average male and female AM rates of 0.20 and 0.14, respectively. In 1981, these rates were 0.27 and 0.20, respectively [6], clearly showing that AM has decreased by 6% in the past 35 years. Likewise, multiple disparities in AM were noted in all categories. In the bivariate analysis, the AM in both sexes was observed to be more than 2 times higher in politically unstable countries and in Sub-Saharan Africa as compared to politically stable and non-African countries. Likewise, female AM was about 3 times higher in countries that have not achieved UHC. A linear regression was performed among 3 independent variables: UHC, economic level, and political stability. We did not include the remaining variables in regression model because any country or continents and religion, as a whole, could not predict AM. Thus, these two variables did not have theoretical foundation; as a result, it may lead to an interpretation bias after the estimation of the standard coefficient. Having a health care system without UHC, political instability, and poor economic status could predict high AM in men and in women (Table 5).

By sex, there was 6% higher AM in men as compared to women. The Centre for Disease Control and Prevention (CDC) showed that male mortality was up to 40% higher than female mortality [31]. Other previous studies also showed that mortality and morbidity was greater in men than in women [32, 33]. A study conducted in the US reveled that due to several risk factors for diseases and injury, male AM was higher than female AM [34], which is a similar finding to that observed in the present this study.

There has been substantial research on AM due to risk and medical factors (burden of disease and lifestyle related factors like smoking, drinking, food habits, exercise, gender, etc.) but not on the structural and intermediate factors (political situation, economic capacity, or health policy). In our bivariate analysis, AM in both sexes was higher in Sub-Saharan Africa, politically unstable and poor countries, countries with traditional religion, and those with a health care system without UHC. Some previous studies have reported similar conclusions. AM is significantly higher in black people, and people with poor socioeconomic status in South Africa [35]. Political instability has been found to impact individuals in multiple ways [36], and such impacts have long term effects on health as well [37]. However, in the present study, we could not identify its exact impact on AM. A study performed by Li Quwan explored 84 countries and concluded that political instability and armed conflict immediately increased the male AM rate, and increased the female AM rate as a lingering effect [21]. A book edited by Denial H Levine noted that there was a substantial effect on youth and adults due to political and religious conflict in Latin America [38]. In 2013, mortality in Gaza showed that 5% adult deaths were related to war injuries and male adult deaths were 50% higher than those in females [39]. Due to the long civil war and its proxy effect, Sierra Leone showed a less than 5% reduction in AM in both sexes over the past 35 years [40], despite being a country rich in natural resources and having an HIV/AIDS prevalence of only about 1%.

The present study showed that the AM rate for males in Africa and North America was 0.31 and 0.10, and that for females was 0.26 and 0.06, respectively (Tables 3 and 4). The world mortality report 2013 showed that the male AM rate for Africa and North America was 0.31 and 0.12, and that for females was 0.27 and 0.07, respectively [41].This trend is similar to that reflected by our results. By economic grading, the male AM rate for the low income and high-income OECD countries was 0.31 and 0.10, respectively. Similarly, the female AM rate was 0.28 and 0.05, respectively. The World Mortality Report further reported that the AM rate for men for the least developed and highest income countries was 0.27 and 0.16, respectively. Likewise, the same was 0.23 and 0.07, respectively, for females. These results support the findings of the present study.

In Latin America, health-system reforms have produced a distinct approach to UHC, underpinned by the principles of equity, solidarity, and collective action to overcome social inequalities [42]. As a result, there has been a reduction in all kinds of mortality as well as in AM. A cross-country study by Moreno-Serra concluded that a broader health coverage generally leads to better access to necessary care and improved population health, particularly for poor people, and that it profoundly reduces the mortality rate [43].

According to our multivariate analysis, political stability, high economic status, and UHC were found to be powerful predictors (β > 0.41) of low AM in both sexes. A systematic analysis by Xing Le Feng et al. in 2012 showed that political factors, and health program and intervention were the second and third powerful predictors for a reduction of under 5 mortality [44] and would be similar influence in adult mortality. Observing the scenario in Uganda since the past 25 years, political instability created a health crisis, increased the mortality, reduced economic productivity, and seriously affected the health care system. Failure of the health system, and the resultant low health service coverage and high rates for all types of mortality in conflict and fragile states was reported by the United States Institute for Peace [45]. Similarly, MH Brenner concluded that economic growth was determinant factor for the decline in types of mortality in the twentieth century in the United States [46]. Economic status simultaneously influences the health status, morbidity, and mortality in all age groups and in both sexes. There are no consistent results regarding the influence of economic status on male and female mortality. However, our result showed that economic strength would have twice as much impact on the mortality rate of adult women than that of men (Table 5). A study conducted in Hungary revealed similar results, reporting that the socioeconomic status of women was more significantly associated with middle age mortality than it was in men [47]. A study conducted in 75 countries showed that excessive adult deaths due to cancer were observed in non-UHC countries [48]. The WHO mortality database showed that South Korea, Taiwan, Hong-Kong, and Tunisia experienced significantly reduced AM in men and women after the adoption of the UHC [40]. There are no similar comparisons of AM and UHC, but it is known that UHC accelerates preventive and promotive health programs like vaccination, sanitation, maternal and child health, nutrition, health education, and other related programs so that the adults could be strong since childhood. More importantly, with UHC, treatment of diseases and illnesses without individual financial burden is also assured, which explicitly reduces mortality.

## Conclusions

Adult mortality has been neglected and structural and intermediate factors have been undermined. Globally, adult mortality has not been significantly reduced; it has been shifted from one place to another. Existing policies are not sufficient to address properly. The essence of Sustainable Development Goal (SDG) provides the way to reduce the inequality and stratification with full resources so that the structural causes of AM would significantly reduce [49]. In conclusion, without political stability, there is poor chance of economic growth and better health system, without which, the adult survival is poor, and the socioeconomic development is hampered. In addition, national health and public policies should be formulated considering this situation. Thus, our study is more applicable for policy makers, researchers, international organizations, and interest groups. Being a single measurement study, these findings do not represent disparity within the countries and at the local level. More importantly, there are different dimension of social aspects like social bond, family structure, ethnic majority, political ideology etc. are there but there is no country’s average value to analyze by data. This study pursues to make some index in other social aspects by country. After this, more area will be analyzed. So, generalization of this findings should be done carefully. There is a further need for a longitudinal study, and an analysis should be conducted at the national and sub-national levels, on multiple aspects of adult characteristics.

## Abbreviation

AM: Adult mortality
MDG: Millennium development goal
OECD: Organization economic co-operation and development
SDG: Sustainable development goal
UHC: Universal health coverage
UN: United nations
